# Microstructure of Neutron-Irradiated Al_3_Hf-Al Thermal Neutron Absorber Materials

**DOI:** 10.3390/ma18040833

**Published:** 2025-02-14

**Authors:** Donna Post Guillen, Janelle Wharry, Yu Lu, Michael Wu, Jeremy Sharapov, Matthew Anderson

**Affiliations:** 1 Idaho National Laboratory, 995 University Blvd., Idaho Falls, ID 83401, USA; michael.wu@inl.gov (M.W.); jeremy.sharapov@inl.gov (J.S.); matthew.anderson2@inl.gov (M.A.); 2 Department of Mechanical Science and Engineering, The Grainger College of Engineering, University of Illinois Urbana-Champaign, Urbana, IL 61801, USA; jpw@illinois.edu; 3 Center for Advanced Energy Studies, Boise State University, 997 MK Simpson Blvd., Idaho Falls, ID 83401, USA; yulu@boisestate.edu

**Keywords:** metal matrix composite, thermal neutron absorber, aluminum–hafnium alloys, hafnium aluminide, transmission electron microscopy, energy-dispersive X-ray spectroscopy

## Abstract

A thermal neutron-absorbing metal matrix composite (MMC) comprised of Al_3_Hf particles in an aluminum matrix was developed to filter out thermal neutrons and create a fast flux environment for material testing in a mixed-spectrum nuclear reactor. Intermetallic Al_3_Hf particles capture thermal neutrons and are embedded in a highly conductive aluminum matrix that provides conductive cooling of the heat generated due to thermal neutron capture by the hafnium. These Al_3_Hf-Al MMCs were fabricated using powder metallurgy via hot pressing. The specimens were neutron-irradiated to between 1.12 and 5.38 dpa and temperatures ranging from 286 °C to 400 °C. The post-irradiation examination included microstructure characterization using transmission electron microscopy (TEM) and energy-dispersive X-ray spectroscopy. This study reports the microstructural observations of four irradiated samples and one unirradiated control sample. All the samples showed the presence of oxide at the particle–matrix interface. The irradiated specimens revealed needle-like structures that extended from the surface of the Al_3_Hf particles into the Al matrix. An automated segmentation tool was implemented based on a YOLO11 computer vision-based approach to identify dislocation lines and loops in TEM images of the irradiated Al-Al_3_Hf MMCs. This work provides insight into the microstructural stability of Al_3_Hf-Al MMCs under irradiation, supporting their consideration as a novel neutron absorber that enables advanced spectral tailoring.

## 1. Introduction

Fast reactor technologies have garnered significant attention in recent decades due to their potential role in achieving a closed nuclear fuel cycle and minimizing nuclear waste. These advanced Generation IV reactors utilize a fast neutron spectrum (energies > 1 MeV), meaning that fissions can occur without moderating neutrons into a thermal neutron spectrum (energies ~0.025 eV) like current light water reactors (LWRs) [1]. By operating with a fast spectrum, fast reactors are designed to recycle spent fuels, extracting a larger portion of their energy content and reducing the amount of nuclear waste generated [1]. Additionally, fast reactors can provide a secure means for the disposal of weapons-grade fissile nuclear materials [2]. Although the development and deployment of fast reactors have faced numerous technical and political challenges [3], there is growing interest in fast reactor technologies driven by global pressures to minimize nuclear waste and expand nuclear energy production [4]. Thus, there is an emergent research need for a fast flux testing facility for the development and qualification of fast reactor fuels and materials. But, given the widespread availability of uranium for conventional LWRs and the associated infrastructure, fast reactors have not been operational in the United States since 1994. As a result, much of the fast flux irradiation testing for the development of Generation IV reactor fuels and materials has been conducted abroad. The development of fast reactor technologies and the establishment of a fast flux irradiation testing facility in the United States represent efforts to advance nuclear energy capabilities, enhance fuel cycle sustainability, and allay concerns regarding nuclear waste disposal.

To address this situation, the Idaho National Laboratory’s Advanced Test Reactor (ATR), a 250 MW thermal spectrum LWR, has been identified as a potential site for the development of a fast flux irradiation testing facility within the United States [5]. A concept has been proposed that aims to simulate a fast flux environment within a LWR by surrounding experimental capsules with uranium silicide booster fuel to augment the neutron flux and provide an absorber block to filter the thermal neutrons from reaching the test specimens [6]. This neutron filtering is accomplished using a thermal neutron absorbing metal matrix composite (MMC) material composed of Al_3_Hf intermetallic particles embedded in an aluminum (Al) matrix. The Al_3_Hf intermetallic particles in the MMC have a large cross section for thermal neutron capture and experience significant heating during irradiation [6,7]. Meanwhile, the Al matrix effectively conducts this heat to adjacent coolant channels to effectively manage thermal loads and prevent overheating of the materials [8].

**Figure 1:**
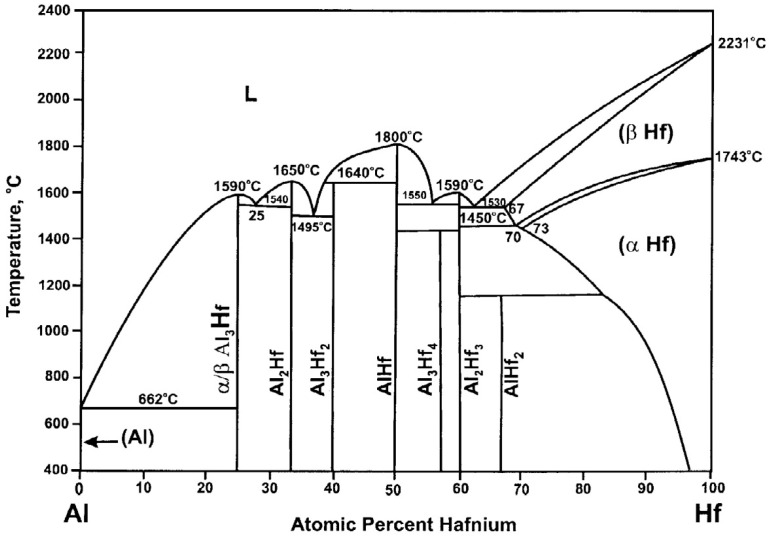
Hf-Al phase diagram, reprinted from [15] with permission from Elsevier.

Alloying with hafnium (Hf) improves the high-temperature (i.e., ≳600 °C) properties of Al [9], thus increasing its suitability for fast flux applications. As seen in Figure 1, the Hf-Al system exhibits a rich variety of intermetallic phases [10]. Al_3_Hf is a Group 4 trialuminide that is a castable, precipitation-strengthened, thermally stable Al alloy [11]. This binary intermetallic crystallizes with a body-centered tetragonal (D0_22_) structure, where each Hf atom is coordinated with 12 Al atoms [12]. Reducing the lattice parameter mismatch between the Al_3_Hf intermetallic particle and the Al matrix is desirable for minimizing the interfacial free energy. Knipling et al. [13] reported the lattice parameters and corresponding mismatch of Al_3_Hf with Al, as shown in Table 1. A concentration of 7 at% Hf (28.4 vol% Al_3_Hf) in the composite material is considered optimal for achieving a fast neutron flux of 10^15^ n cm^−2^ s^−1^ and a fast-to-thermal neutron ratio of 40 within the test capsule [14].

Al-Hf alloys and composites have not been widely studied in nuclear environments, even though Al and Hf materials are currently used within the ATR and have been used for decades with great success—the ATR fuel is clad with Al alloy 6061 and the outer shim cylinders are made from Hf [16]. However, the irradiation behavior of these materials has not been widely explored. In one study by Sturcken [17], commercial Al alloys 1100 and 6063 were irradiated to 20 and 45 displacements per atom (dpa) with >0.2 MeV neutrons at ~112–117 °C. Although precipitation of transmuted Si occurred, the alloys were relatively resistant to swelling, experiencing only 0.22–0.32% swelling. The swelling resistance of Al was also demonstrated in a transmission electron microscopy (TEM) in situ study by Birtcher et al. [18], who conducted 200 keV Xe ion irradiation on thin Al foils that were pre-implanted with 3 keV He ions. Under Xe irradiation, the original He bubbles coalesced into larger bubbles with energetically favorable spherical shapes. But upon continued Xe ion irradiation, Al surface sputtering occurred, causing some bubbles to disappear (i.e., lost to the free surface). The bubbles that remained in the material shrank due to irradiation-induced displacement of He out of the bubbles while the bubbles maintained their equilibrium pressure. The fundamental point defects generated in Al by irradiation have been theorized based on resistivity recovery studies following electron [19,20] and proton [21] irradiation. These studies have suggested that vacancies and two interstitial types are the stable point defects formed and have shown that solute atoms, especially H, can influence the activation energies. Mechanically, Sturcken’s [17] commercial Al alloys 1100 and 6063 experienced irradiation-induced embrittlement, but the hardening and reduction in ductility were significantly lower than in Mg alloys irradiated to similar conditions. Neutron irradiation appreciably increases the formation of dislocations in metals by creating point defects, such as vacancies and interstitials. When neutrons collide with atoms in the metal lattice, they displace atoms, generating these point defects. These defects then diffuse and cluster together to form dislocation loops, which raise the overall dislocation density within the material. This process, known as “irradiation hardening”, makes the metal harder and less ductile since the new dislocations impede the movement of existing ones.

Significant knowledge gaps remain relative to the irradiation effects on Al and Al_3_Hf-Al MMCs. The microstructural stability of these materials under irradiation is critical to their long-term use as thermal neutron absorbers for fast flux testing setups. The present study aims to provide a foundational and systematic understanding of irradiation effects on Al_3_Hf-Al MMCs. Here, we will present the results from a neutron irradiation experiment designed to systematically vary irradiation temperature and dpa. TEM characterization reveals defect microstructures, specifically dislocation loops, and inter-phase structural evolution and chemistry. An automated computer vision-based dislocation recognition tool is implemented to identify dislocation lines and loops in TEM images. Automated analysis of dislocations has the potential to be significantly more efficient, accurate, and repeatable than manual analysis. Moreover, it can easily adapt to process large amounts of data produced by high-throughput characterization methods. Overall, the results show promise for the reasonable irradiation performance of Al-Al_3_Hf composites as advanced neutron absorber materials that can enable spectral tailoring in a LWR to be used for qualification of fast reactor fuels and materials.

## 2. Materials and Methods

**Figure 2:**
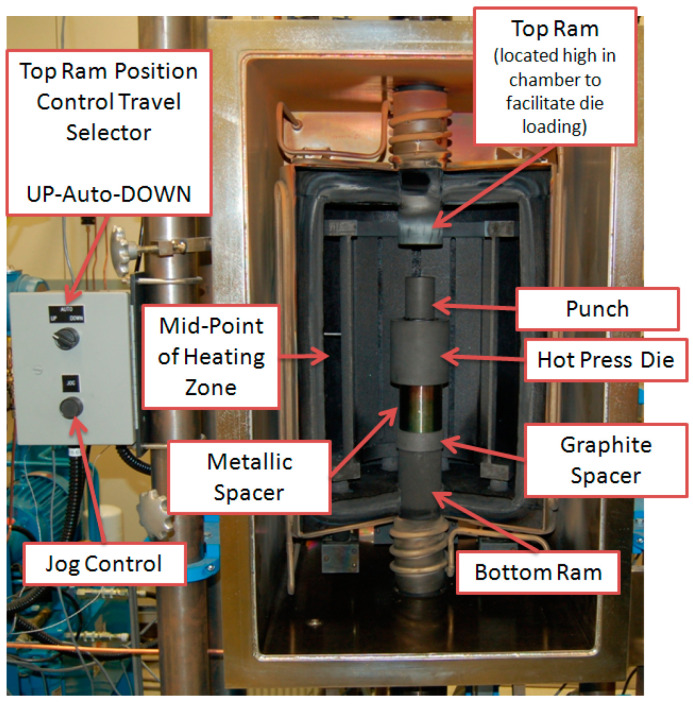
Hot press used for sample fabrication.

The Al_3_Hf intermetallic alloy was produced via centrifugal casting of Hf bar stock (Alfa Aesar, Haverhill, MA, USA) and Al granules, which were then ground into powder using a mortar and pestle. The phase of the alloy was examined and confirmed using X-ray diffraction. The powder was sonically sieved in an argon atmosphere glovebox to retain particles less than 38 µm in size and mixed at various volume fractions with high-purity Alcoa 101 powder (Alcoa Corp., Pittsburgh, PA, USA). The optimal Al_3_Hf volume fraction for the absorber block, determined through neutronics and thermal evaluations, was calculated to be 28.4 vol% [14]. Consequently, this material volume fraction was the focus of this study. Due to the potential hazards associated with fine aluminum powders, the powders were loaded into the mixing vial within the glovebox. Mixing was performed using a Turbula^®^ mixer (WAB US Corp., Allendale, NJ, USA). After mixing, the powder was uniaxially hot pressed into pucks to form the Al_3_Hf-Al MMCs (Figure 2) [22]. Electron backscatter diffraction observations revealed that the majority of the Al_3_Hf and Al grains in the final sample had diameters of less than 10 µm [23].

**Figure 3:**
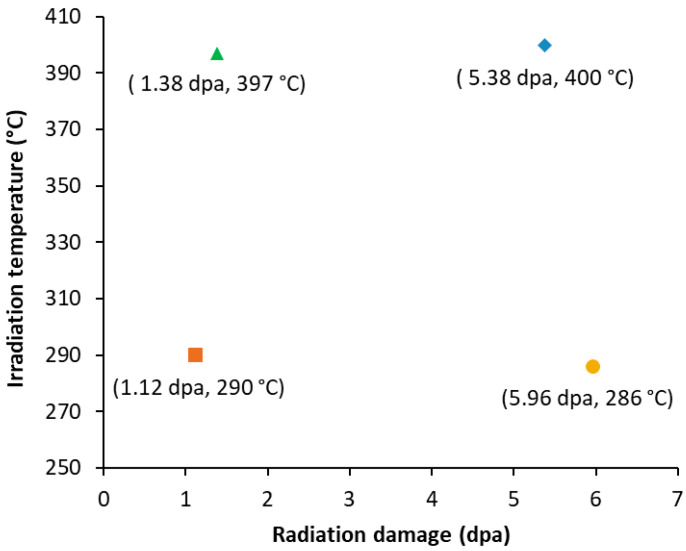
Irradiated specimens examined in this study with as-run calculated temperature and dpa.

Specimens of the Al_3_Hf-Al MMCs were machined from the pucks into discs with a 3 mm diameter and 300 µm thickness. The specimens were contained within drop-in capsules backfilled with an inert gas mixture designed to control the temperature during irradiation. The capsules were housed in an irradiation assembly in the ATR inner A positions and irradiated to achieve four temperature–dpa conditions. The experiment was designed to achieve target radiation damage of 1 ± 0.2 dpa and 3 ± 0.2 dpa at temperatures of 300 ± 50 °C and 400 ± 50 °C to allow for systematic understanding of radiation damage and temperature dependence [24]. The specimen temperatures and dpa were tailored by the axial placement of the specimens within capsules in the test train assembly. The samples housed in the 1 dpa capsules were irradiated in the A-7 and A-8 positions during Cycle 164A, whereas the 3 dpa capsules were irradiated in the A-6, A-7, and A-8 positions during Cycles 164A, 164B, and 166A. The effective full power days of irradiation were 54.9 for the 1 dpa capsules and 187.8 for the 3 dpa capsules. The as-run calculated temperatures and dpa for the specimens selected for this study are shown in Figure 3 [25]. These Al_3_Hf-Al MMCs were irradiated at higher temperatures compared to previous experiments [26]. A Monte Carlo neutron particle [27] full core physics model for the ATR was used to calculate the as-run heat rates, flux and fluence values, and dpa for each of the test specimens. Dpa is a damage-based exposure unit used to quantify radiation damage by representing the number of atoms displaced from their normal lattice sites due to energetic particle bombardment, providing a standard measure for neutron- and gamma-induced radiation damage in materials [28]. The fluence data were gathered by integrating the flux values over their respective cycle times and adding the fluence values from each cycle to provide the total neutron fluence. The total neutron fluences were 2.85 × 10^20^ (1.12 dpa, 290 °C), 1.52 × 10^22^ (5.96 dpa, 286 °C), 3.57 × 10^20^ (1.38 dpa, 397 °C), and 1.40 × 10^22^ n/cm^2^ (5.38 dpa, 400 °C) [29].

The irradiated specimens had a surface oxide layer, which was a consequence of using water to decontaminate radioactive particles from the specimens during disassembly of the capsules in the hot cell. This surface oxide layer was polished off before extracting a TEM lamella from each specimen using the focused ion beam (FIB) lift-out technique using a FEI Quanta 3D dual-beam FIB/SEM (Thermo Fisher Scientific, Hillsboro, OR, USA). Both sides of each lamellae were milled with 2 kV Ga^+^ as a final step to minimize the damage from FIB. Then, the lamellae surfaces were cleaned using a Fischione Model 1040 Nanomill (E.A. Fischione Instruments, Inc., Export, PA, USA) with a low beam energy of 600 eV to further remove the Ga^+^ damage layers from the FIB process. An unirradiated control specimen was also prepared for TEM using the same FIB and low-energy ion milling processes.

High-resolution TEM characterization was performed with a FEI (now ThermoFisher) Tecnai G2 F30 Scanning TEM (STEM) (Thermo Fisher Scientific, Waltham, MA, USA). Brightfield (BF) STEM images were acquired to visualize the coherency of the interface between the particles and the matrix. Energy-dispersive X-ray spectroscopy (EDS) was applied to study the chemical composition evolution across the interface. On-zone axis BF-STEM images were used to image and quantify dislocation loops and dislocation lines within the lamellae following common practice for irradiated alloys [30]. The thicknesses of the TEM lamellae were measured using the electron energy loss spectroscopy (EELS) technique.

**Figure 4:**
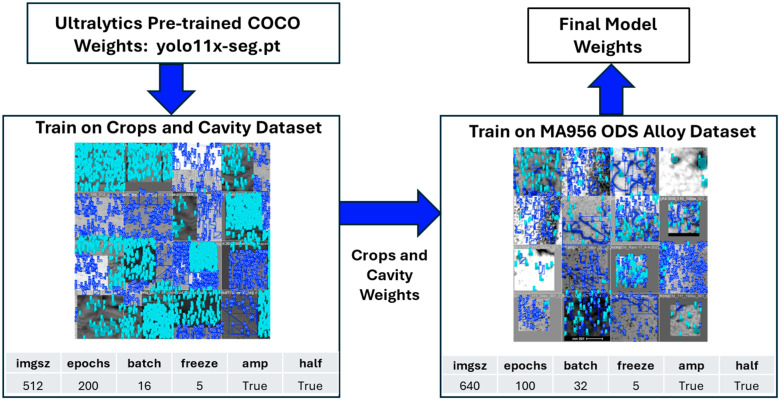
Transfer learning process for quantifying dislocation defects. The model was initially trained on crops and a synthetic cavity dataset. The resulting weights were then fine-tuned using MA956 ODS alloy images to obtain the final model weights.

YOLO11, the latest version of the YOLO (You Only Look Once) family of object detection algorithms, is used within the broader field of computer vision [31]. The Ultralytics YOLO leverages transfer learning to develop an efficient dislocation defect quantification tool, requiring only a small set of annotated micrographs for training [32]. Typically, achieving optimal training results with YOLO11 requires a substantial dataset, with over 1500 images per class [33]. To address this, the YOLO11 segmentation model (YOLO11x-seg.pt) was initially trained on the Common Objects in Context (COCO) dataset, which comprises over 330,000 annotated images across 80 object categories, though it does not include any TEM images [34]. Figure 4 illustrates the training procedure along with the settings used to tune the models, where *imgsz* defines the target dimensions for resizing all images during model training; *epochs* indicate the total number of times the entire dataset is passed through the network during training; *batch* size represents the number of training images processed before the model’s weights are updated; *freeze* specifies the number of initial layers whose weights remain unchanged during training; *amp* stands for automatic mixed precision, optimizing performance and memory usage; and *half* enables inference using 16-bit (half precision) floating-point numbers [35]. The COCO dataset served as the primary training set for the first stage of transfer learning [36]. The pre-trained COCO weights were then used to train on a crops [37] and cavity [38] image database for 200 epochs, resulting in a set of intermediate weights. These optimal weights, determined from the epoch with the highest validation metrics, were further fine-tuned using a MA956 oxide dispersion strengthened (ODS) alloy dataset for 100 epochs. To augment the dataset, five defect-annotated MA956 ODS alloy images were cropped into 50 smaller images, with 45 images used for training and 5 images used for validation. This entire training process took approximately 30 min using an NVIDIA A100 40 GB GPU. Validation of MA956 ODS defects involved comparing the ground truth against the prediction masks for two noisy BF-STEM images with overlapping defects. The F1 scores were 0.60 for lines and 0.59 for loops in the first image, and 0.57 for lines and loops in the second image [32].

This method is a significant advancement over other automated image analysis approaches that require an extensive database of manually annotated images for training, such as that implemented by Li et al. for irradiated steels [39]. The object detection technique implemented here was trained to identify and segment both dislocation lines and loops simultaneously, focusing on extracting quantitative defect structure and distribution while only being trained on five manually annotated images.

## 3. Results and Discussion

### 3.1. Dislocation Defect Detection and Quantification

**Figure 5:**
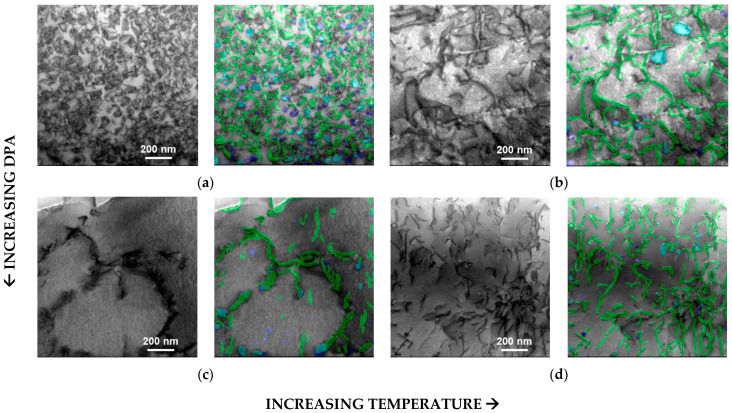
On-zone axis BF-STEM images in the Al_3_Hf showing dislocation lines and loops under various irradiation dpa values and temperatures. For each pair of images, the left image is the original BF-STEM image, and the right image shows the YOLO11-detected lines and loops overlaid (green mask represents dislocation lines, blue mask represents loops, and teal mask represents overlap between lines and loops). Magnification of all images is 96 kx. (**a**) 1.12 dpa, 290 °C. (**b**) 1.38 dpa, 397 °C. (**c**) 5.96 dpa, 286 °C. (**d**) 5.38 dpa, 400 °C.

**Figure 6:**
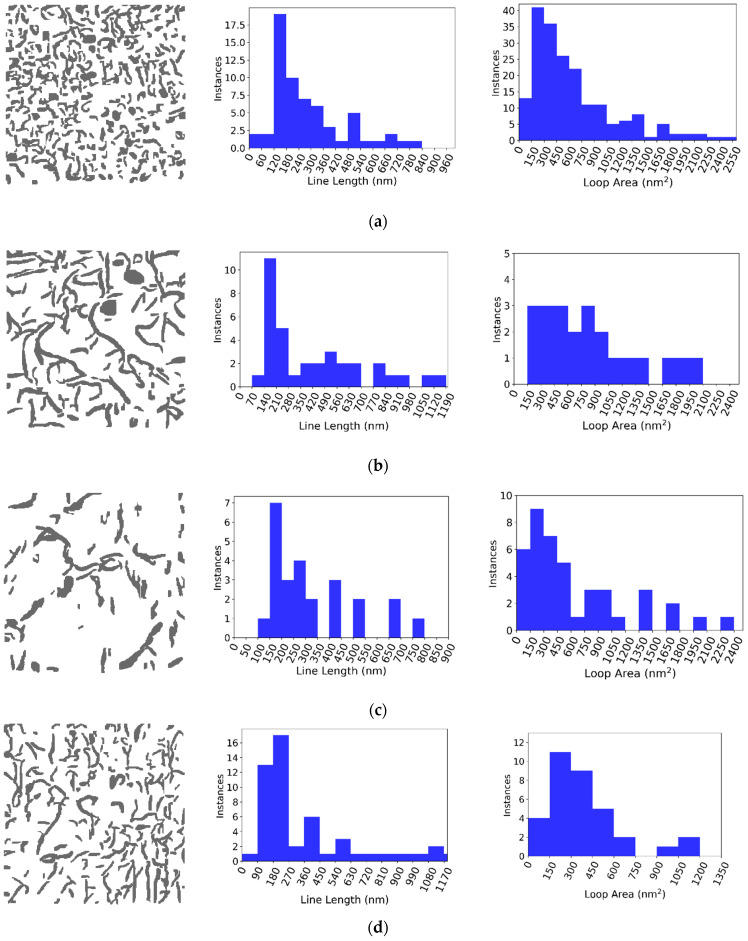
Histograms of line lengths and loop areas corresponding to the prediction mask on the left. (**a**) 1.12 dpa, 290 °C. (**b**) 1.38 dpa, 397 °C. (**c**) 5.96 dpa, 286 °C. (**d**) 5.38 dpa, 400 °C.

Figure 5 shows the results of the YOLO11 object detection algorithm for a BF-STEM image from each of the four different irradiation conditions. The on-zone axis BF-STEM images show the defects inside of a Al_3_Hf grain. The images are juxtaposed to facilitate comparison. For each condition, the left image is the original BF-STEM image, and the right image shows the YOLO11-detected dislocation lines and loops. The green mask indicates dislocation lines, the blue mask indicates dislocation loops, and the teal mask indicates regions of overlap between dislocation lines and loops. Table 2 tabulates the number of dislocation lines and loops detected by YOLO11, along with the defect dislocation density for the images in Figure 5a–d. The lamella thicknesses measured by EELS and zone axes for the BF-STEM images are also listed. Figure 6 illustrates the detected dislocation lines and loops in the BF-STEM images shown in Figure 5 with corresponding histograms of line lengths and loop areas. For all irradiation conditions, the largest population of lines falls in the 10–300 nm range. The line length histograms for all the irradiation conditions are positively skewed, with the population of lines generally decreasing for longer lines. The loop area histograms are similarly positively skewed, showing the highest population for loops less than ~600 nm², although a few larger loops are present.

The on-zone axis BF-STEM images in Figure 5 show dislocation loop and line networks in the Al matrix that vary in relative population, size, and number density across irradiation conditions. At the lowest dpa and temperature, 1.12 dpa at 290 °C, the network is comprised almost exclusively of dislocation loops, as shown in Figure 5a. With increasing irradiation temperature, 1.38 dpa at 397 °C, a few large dislocation loops remain (~50–150 nm in diameter) amongst a relatively high density of dislocation lines formed by loops growing too large and subsequently unfaulting (Figure 5b). With increasing dpa, nearly all the dislocation loops completely unfaulted into dislocation lines, and dislocation line recovery occurred for the 5.96 dpa, 286 °C and 5.38, 400 °C conditions, respectively (Figure 5c,d). At the highest dpa and temperature, 5.38 dpa at 400 °C, the dislocation loops almost completely unfaulted into dislocation lines, and dislocation line recovery occurred (Figure 5d).

The observed dislocation loop and network evolution is reasonable given that the target irradiation temperatures of 300 °C and 400 °C represent homologous temperatures (T_h_) of 0.61 and 0.72, respectively. That is, even at the lowest dpa–temperature condition, the loops are already large. Any increase in dpa and/or temperature will lead to rapid loop growth resulting in loop destabilization and unfaulting into lines. Given the high T_h_, dislocation recovery is also kinetically favorable, especially since the specimens are held at temperature for 1–2 years during irradiation. This is confirmed by comparing the loop densities in Al_3_Hf to that in higher-melting-temperature alloys irradiated in the same irradiation campaign. For example, low-alloy steel SA508 irradiated to 0.69 dpa at 286 °C (T_h_ of 0.33) and 0.95 dpa at 386 °C (T_h_ of 0.39) has a dislocation loop number density 1–2 orders of magnitude greater than that observed here for Al_3_Hf [40,41]. Similarly, Ni-based Alloy 625 irradiated to ~1.05 dpa at 386 °C (T_h_ of 0.41) has a loop number density of 1.1–1.4 × 10^22^ m^−3^, two orders of magnitude greater than observed for Al_3_Hf [42]. Indeed, when identical Alloy 625 specimens are ion irradiated at a higher T_h_ of 0.48 (500 °C) to 50–100 dpa, the loop number densities decrease to 2.4–6.2 × 10^−21^ m^−3^ [43], partway between the loop densities formed at a T_h_ of 0.41 and T_h_ of 0.61–0.72.

### 3.2. Particle–Matrix Interface Evolution

**Figure 7:**
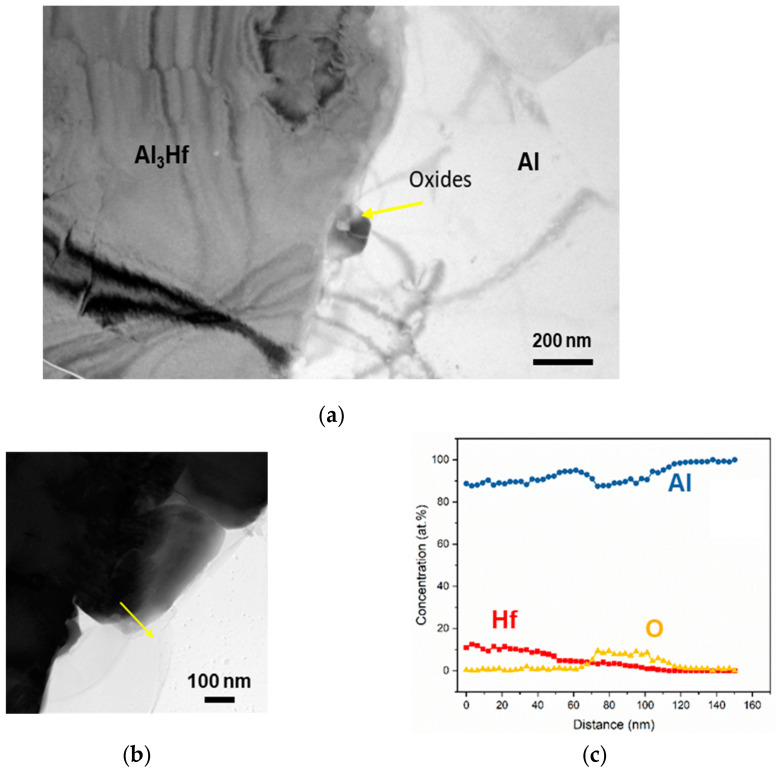
Unirradiated Al–Al_3_Hf interface shown in (**a**) BF-STEM image with residual oxide along interface imaged at a magnification of 15 kx, and (**b**) higher-magnification BF-STEM image with arrow indicating direction of (**c**) corresponding EDS linescan revealing Hf, Al, and O concentration profiles.

BF-STEM of the unirradiated Al-Al_3_Hf interface (Figure 7) revealed a ~30 nm oxide thickness between the two phases. This oxide is likely an artifact of the powder processing, since powder particles are known to retain residual oxides after hot pressing [44]. At the phase boundary shown in Figure 7b, the Al_3_Hf particle is diffused into the Al matrix, as confirmed by the EDS linescan.

**Figure 8:**
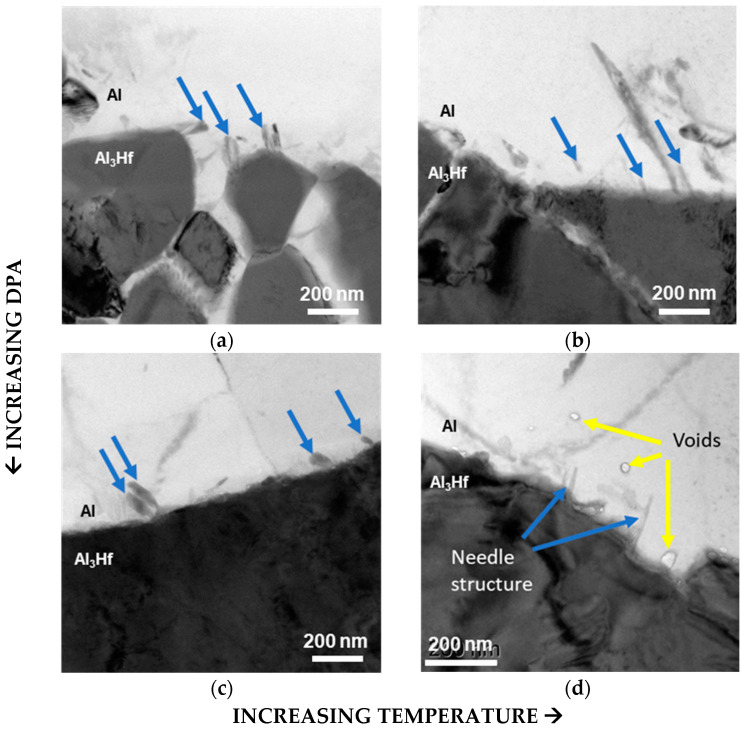
BF-STEM images of Al–Al_3_Hf interface showing needle-like structures forming in the Al matrix (indicated by blue arrows) under the following conditions: (**a**) 1.12 dpa, 290 °C, (**b**) 1.38 dpa, 397 °C, (**c**) 5.96 dpa, 286 °C, and (**d**) 5.38 dpa, 400 °C. Voids are observed in the Al matrix (indicated by yellow arrows) only in the 5.38 dpa, 400 °C sample.

**Figure 9:**
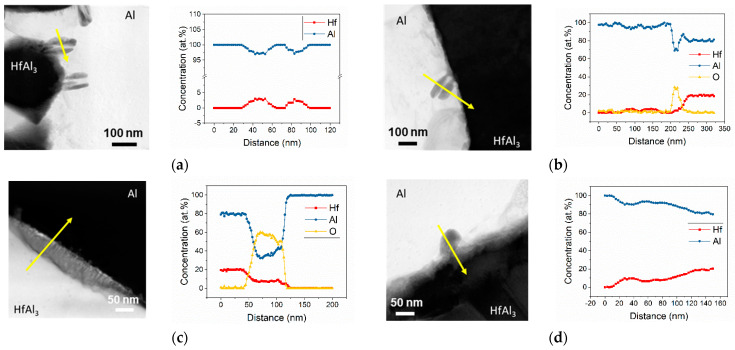
TEM EDS line scans (indicated by yellow arrows) and Hf, Al, and O composition profiles across irradiation-induced needle-like structures in Al matrix under the following conditions: (**a**) 1.12 dpa, 290 °C, (**b**) 1.38 dpa, 397 °C, (**c**) 5.96 dpa, 286 °C, and (**d**) 5.38 dpa, 400 °C.

But, after irradiation, BF-STEM imaging showed that needle-like structures formed within the Al matrix along the particle–matrix boundary (Figure 8). The aspect ratios of the needles appeared larger (i.e., longer and thinner) in the ~400 °C irradiation conditions than in the ~300 °C irradiation conditions, which could be due to the accelerated kinetics at the higher temperature. The needles did not appear to exhibit significant evolution as a function of dpa. Corresponding TEM EDS line scans indicated that the needle-like structures were Hf-rich, and there was oxide present at the interface (Figure 9). In the 5.96 dpa, 268 °C condition, an EDS line scan was taken across the particle–matrix interface rather than across the needles (Figure 9c), revealing a thicker oxide layer than that prior to irradiation.

Void swelling has a significant impact on the service performance of materials used in the nuclear field, as it can greatly affect the material’s mechanical integrity [45,46,47] and thermal conductivity [48]. In this study, voids were observed only in Al_3_Hf-Al irradiated under the highest dpa–temperature condition (5.38 dpa and 400 °C). Although a few studies have evaluated irradiation-induced microstructure evolution in Al and Al alloys, these reports consistently suggest that these materials are relatively resistant to void swelling until high doses. Unfortunately, due to the age of these studies, the doses are reported in neutron fluence, so drawing a direct comparison across dpa levels is not straightforward [17,49,50]. The voids primarily appeared in the vicinity of the Al_3_Hf–Al interface, suggesting that void formation induced by irradiation is likely to compromise the material’s integrity at this interface. Additionally, the voids are highly likely to affect thermal conductivity, which in turn impacts the material’s function as a thermal neutron-absorbing MMC. Therefore, the irradiation-induced voids need to be further assessed before Al_3_Hf can be applied in reactors. However, the current data are still very limited. To better understand the void swelling effect on Al_3_Hf particles in the aluminum matrix, more studies under higher irradiation temperatures and higher dpa values are needed.

## 4. Conclusions

A thermal neutron-absorbing MMC consisting of Al_3_Hf particles in an aluminum matrix was developed to filter thermal neutrons and create a fast flux environment for material testing in mixed-spectrum nuclear reactors. The MMC combines neutron absorbing particles with a highly conductive metal matrix, enabling effective conductive cooling of the heat generated from thermal neutron capture by hafnium. These Al_3_Hf-Al MMCs were fabricated using powder metallurgy via hot pressing and then neutron irradiated to dpa values between 1.12 and 5.38 dpa at temperatures ranging from 286 °C to 400 °C. The key outcomes of this study are enumerated below:

1. An open-source object segmentation method was developed to automate the detection and quantification of dislocation defects. This versatile methodology allows for its use on diverse datasets and materials different from those on which it was trained. The code is user-friendly and does not require extensive programming knowledge. Segmentation masks facilitate the determination of metrics such as line lengths, loop areas, number of lines, number of loops, and dislocation defect density. Since developing a database of manually annotated images is quite time-consuming, the application of transfer learning can facilitate high-throughput analysis of TEM images.
  The results indicate that neutron irradiation significantly impacts the microstructure of Al_3_Hf-Al composites, with observable defect formations such as dislocation loops and lines. For low-temperature irradiation with low dpa values, many dislocation loops were observed within the Al_3_Hf particles. As the dpa increased to 5.96, the majority of the defects became dislocation lines, with fewer dislocation loops. For samples irradiated to 1.38 and 5.38 dpa at higher temperatures, dislocation lines were the primary defect, with only a few dislocation loops.
2. Post-irradiation examinations included microstructural characterization using TEM and EDS. We analyzed four irradiated samples and one unirradiated control sample. Oxygen was observed at the particle–matrix interface in all the samples. TEM of the irradiated samples revealed needle-like structures extending from the Al_3_Hf particles into the Al matrix, which were absent in the unirradiated samples. The high-temperature/high-dpa specimen showed voids within the Al matrix.
3. Oxygen segregation at the interface was observed in both the unirradiated and irradiated samples, with irradiation enhancing this segregation. However, due to the limited number of phase boundaries examined, definitive conclusions about the effects of dpa or temperature on oxygen segregation could not be drawn

This study provides insights into the microstructural stability of these MMCs under irradiation, supporting their potential as novel neutron absorbers for advanced spectral tailoring in LWRs and playing a crucial role in the qualification of fast reactor fuels and materials, enhancing the development of fast flux irradiation testing facilities.

## Figures and Tables

**Table 1:** Reported lattice parameters for Al and Al_3_Hf phases with calculated mismatch at room temperature [13].

Phase	Structure	Lattice Parameters [Å]	Mismatch with Al	Absolute Mismatch
Al	FCC	4.0496	a: −3.87% c: +10.20%	5.98%
Al_3_Hf	D0_22_	a = 3.893 c = 8.925		

**Table 2:** BF-STEM details and predicted total number of dislocation lines, loops, and number densities detected by the YOLO11 algorithm for each irradiation condition.

Dpa	Temperature (°C)	Zone Axis	Lamellae Thickness (nm)	Dislocation Lines (Number)	Dislocation Loops (Number)	Line Number Density (m^−3^)	Loop Number Density (m^−3^)
1.12	290	[001]	68.35	666	194	6.72 × 10^21^	1.96 × 10^21^
1.38	397	[041]	125.88	526	35	2.86 × 10^21^	1.91 × 10^20^
5.96	286	[221]	88.82	315	24	2.43 × 10^21^	1.85 × 10^20^
5.38	400	[221]	106.34	149	46	9.87 × 10^20^	3.05 × 10^20^

## Data Availability

The data supporting the findings of this study are openly available via the Nuclear Science User Facilities’ Nuclear Research Data System, TEM Characterization of Neutron Irradiated Al_3_Hf-Al Composite Specimens: https://doi.org/10.48806/2478162 (2024). The code and models, along with the training, test, and validation datasets, are available at: https://github.com/idaholab/PANDA (accessed on 2 February 2025). The MA956 ODS images used for training are available at: https://doi.org/10.48806/2468645.
